# Proportion and factors associated with fetal adverse birth outcome among mothers who gave birth at Felege Hiwot Comprehensive Specialized Hospital, Bahir Dar city, Northwest, Ethiopia 2019

**DOI:** 10.11604/pamj.2022.42.76.34686

**Published:** 2022-05-27

**Authors:** Bezawit Abeje Alemayehu, Selamawit Lake Fenta, Shumiye Shiferaw Gessesse, Tigist Wubet Gezahegn, Eden Asmare Kassahun, Wondu Feyisa Balcha

**Affiliations:** 1 Department of Midwifery, College of Medicine and Health Sciences, Bahir Dar University, PO. Box: 079, Bahir Dar, Ethiopia

**Keywords:** Adverse birth outcome, proportion, Bahir Dar, Ethiopia

## Abstract

**Introduction:**

fetal adverse birth outcomes are abnormal outcomes such as prematurity, low birth weight, stillbirth, and birth defects. It is the main cause of neonatal and child deaths in the world and is the major public health problem in developing countries including Ethiopia. This study aims to assess the proportion and factors associated with fetal adverse birth outcomes among mothers who gave birth at Felege Hiwot Comprehensive Specialized Hospital, Bahir Dar city, North-west, Ethiopia 2019.

**Methods:**

institution-based cross-sectional study was conducted from March 1- to April 30 in 2019 among 371 delivered mothers. The data were collected by systematic random sampling technique, entered into a computer using Epi data 3.5, and analyzed using Statistical Package of Social Sciences version 23.0. Bivariate and multivariable logistic regression analyses were done to estimate the crude and adjusted odds ratio with a confidence interval of 95% and a P-value of less than 0.05 considered statistically significant.

**Results:**

in this study, the proportion of fetal adverse birth outcome was 33.2%. Mothers who lived in rural area [AOR=4.37, 95% CI=2.44-7.83], < 4 antenatal care visit [AOR=1.91, 95% CI=1.08-3.40], bad obstetrical history [AOR=2.01, 95% CI=1.03-3.93], complication in the antepartum period [AOR=4.32, 95% CI=2.44-7.65], medical illness [AOR=2.44, 95% CI=1.25-4.79], and maternal hemoglobin level < 11 mg/dl [AOR=4.63, 95% CI=2.40-8.93] were significantly associated with fetal adverse birth outcomes.

**Conclusion:**

the proportion of fetal adverse birth outcomes in this research was high. Living in a rural area, the number of antenatal care visits, bad obstetrical history, current pregnancy complications, medical illness, and hemoglobin levels less than or equal to 11 mg/dl were significantly associated with fetal adverse birth outcomes. Getting full service of antenatal care visits and advance in the quality of maternal health services could minimize fetal adverse birth outcomes.

## Introduction

Fetal adverse birth outcomes (FABO) are abnormal birth outcomes which include: preterm birth, low birth weight (LBW), stillbirth, and congenital anomaly. Globally, more than one in ten newborn babies develop FABO [1]. FABO is the major cause of neonatal morbidity and mortality in the world, and also the principal cause of all child deaths. Babies who are born with adverse birth outcomes and who stay alive face a lifetime of infirmity [2]. Worldwide, each year around 210 million women become pregnant, and from this around 35% of pregnancies end in stillbirth, preterm birth, spontaneous or induced abortion, 2.5 million babies die during their first month of life and 2.0 million babies are stillborn [3]. Sub-Saharan Africa has the highest rates of maternal and neonatal mortality worldwide [4]. In Ethiopia, according to the 2016 Ethiopian Demographic and Health Survey report, the rate of perinatal mortality was 33 per 1000 pregnancies [5].

Studies conducted in different countries showed that FABO were significantly affected by extreme maternal age (age ≥ 35 and < 20 years), gestational age, marital status, maternal anemia, mothers who were on oral contraceptives, pre-eclampsia/eclampsia, APH, PROM, history of preterm delivery and abortion, multiple pregnancies, urinary tract infection, lack of formal education, being a merchant and residing in a rural area, maternal body mass index <18 kg/m^2^, inter-pregnancy interval <2 years absence of antenatal care (ANC) visits, history of stillbirth, maternal malaria, gravidity, parity, the onset of labor, previous bad obstetrical history (BOH), hemoglobin level less than 11 mg/dl, type of pregnancy and chronic medical illness like HIV-infected women were significantly associated with adverse birth outcomes [6-22]. Birth outcomes are measures of wellbeing at birth and have enhanced intensely worldwide in the past 40 years. But still, there is a great gap between the outcomes in developing and developed countries [23]. According to the reports of different studies in Ethiopia, FABO ranges from 18.3% to 37.6% and LBW and preterm birth accounts for the majority of FABO [17-22]. By the year 2030, the Sustainable Development Goals (SDG) target is to reduce neonatal deaths to 12 per 1000 live births, and under-five deaths to less than 25 per 1000 live births by eliminating preventable child deaths [24]. Therefore, knowing the proportion of FABO helps to achieve the strategy of the SDG. The objectives were to determine the proportion of FABO among mothers who gave birth at Felege Hiwot Comprehensive Specialized Hospital, Bahir Dar city, North-west, Ethiopia, to identify factors associated with FABO among mothers who gave birth at Felege Hiwot Comprehensive Specialized Hospital, Bahir Dar city, North-west, Ethiopia.

## Methods

**Study design and period:** an institutional-based cross-sectional study design was employed from March 1-May 1, 2019 at Felege Hiwot Comprehensive Specialized Hospital (FHCSH), Bahir Dar city.

**Study area:** the study was conducted at FHCSH in Bahir Dar city. Bahir Dar City is the capital city of the Amhara Region. The city is located approximately 565 km northwest of Addis Ababa, the capital city of Ethiopia. The city has a total population of 518,193 of which 265,156 are females [25]. FHCSH has different departments, and it serves an estimated eight million people residing in urban and rural parts of North West Ethiopia per year. The obstetric ward is the one that gave on average 6480, 540, and 18 delivery services per year, month, and day respectively.

**Source population:** all mothers who gave birth at FHCSH.

**Study population:** all selected mothers who gave birth at FHCSH during the study period.

### Sample size determination

The sample size was calculated using a single population proportion formula by considering the following assumptions: proportion of FABO in Dessie referral hospital was 32.5% [**26**], Zα/2= critical value for normal distribution at 95% confidence level, which is equal to 1.96 (Z value of alpha=0.05) or 5% level of significance (α=0.05) and a 5% margin of error (ω =0.05).


$$Sample\text{ size (n)=}\frac{{\left(Z\alpha /2\right)}^{2}p(1-p)}{d^{2}}$$

N = (1.96)^2^0.325(1-0.325) / (0.05)^2^= 337

The sample size was adjusted by adding a 10% non-response rate, and the final total sample size was 371.

**Sampling procedure and technique:** the eligible mother was selected by using systematic random sampling techniques. The sampling interval (Kth units =984/371 =3) was obtained by dividing the number of monthly mothers who gave birth by the total sample size of the study. The starting unit was selected by using the lottery method among the first kth units. In case the type of pregnancy was multiply, the one was selected by lottery method.

**Dependent variable:** fetal adverse birth outcomes.

**Independent variables:** sociodemographic characteristics: residence, age, marital status, occupation, religion, and ethnicity; obstetric history: parity, pregnancy status, ANC follow-up, the timing of first ANC visit, number of ANC visits, iron supplementation, duration of iron supplementation and history of contraceptive use, type of pregnancy, gestational age, the status of labor, mode of delivery antepartum hemorrhage, pregnancy-induced hypertension, premature rupture of membrane, obstructed labor, Amniotic fluid disorders, and uterine rupture; medical illness: DM, hypertension, heart problem, HIV status, anemia, malaria.

### Operational definitions

**Fetal adverse birth outcomes:** a woman who had at least one of the following; stillbirth, low birth weight, preterm delivery, and visible birth defect [27].

**Stillbirth:** a baby born with no signs of life at or after 28 weeks gestation [26].

**Low birth weight:** any newborn weighing less than, 2500gm at birth [1].

**Preterm delivery:** a neonate that is delivered before 37 completed weeks of gestational age [28].

**Visible birth defects:** any newborn has a defect in his/her body structure [29].

**Bad obstetric history:** previous unfavorable fetal outcome in terms of two or more consecutive spontaneous abortions, history of IUGR, stillbirth, preterm labor, low birth weight, and congenital anomaly [30].

### Data collection tools and procedures

A structured interviewer-administered questionnaire was used to collect the data which were adapted from relevant works of literature and modified to the local context [2,3,6-10,13,16-23,27,29]. Questionnaires were first prepared in the English language, then it was translated into Amharic by an individual who has a good ability in these languages, then retranslated back into English to check the consistency. The questionnaire consisted of sociodemographic characteristics, Reproductive and obstetric characteristics, and the medical history of the mother. A pre-tested structured interviewer-administered questionnaire was used for data collection purposes. The data were collected by three diploma midwives and supervised by one BSc midwife.

### Data quality assurance

Data were collected by trained data collectors, and pre-testing of the instrument was done before the actual data collection. The questionnaire was pre-tested on 5% (19) of the sample. Data collectors and the supervisors were trained for two days by the investigator. After necessary modifications and correction was done to standardize and ensure its reliability and validity, additional adjustments were made based on the results of the pre-test and daily supervision was done.

### Data processing and analysis

The data were entered into Epi data 3.5, edited and cleaned for inconsistencies, missing values, and outliers, then exported to the statistical package of social science 23.0 version for analysis. During analysis, all explanatory variables which have a significant association in bivariate analysis with a P-value < 0.20 were entered into a multivariable logistic regression model to get an adjusted odds ratio (AOR), and those variables with 95% of confidence intervals (CI) and a P-value of < 0.05 was considered as statistical significance with FABO. The multicollinearity test was done using the variance inflation factor, and no collinearity exists between the independent variables. The model goodness of the test was checked by Hosmer- Lemeshow goodness of the fit test, and its P-value was > 0.5. Frequency tables, figures, and descriptive summaries were used to describe the study variables.

### Ethical approval and consent to participate

Ethical clearance was obtained from the Institutional Review Board of Bahir Dar University College of medicine and health science (CMHS/ IRB 01-008). A formal letter was also obtained from FHCSH medical director. The purpose of the study was explained to each mother. At the time of data collection, written consent was obtained from each study participant for those ages greater than 18 years and from parents/guardians for those ages less than 18 years. All respondents were assured that the data would not have any negative consequences on any aspect of their life.

**Data availability:** all related data have been presented within the manuscript. The data set supporting the conclusion of this article is available from the corresponding author upon reasonable request.

## Results

### The socio-demographic characteristics of the mothers

A total of 371 mothers participated, with a response rate of 100%. The mean age of the mothers was 28 years with a standard deviation of + 5.38 and nearly three fourth (74.1%) of the mothers were found in the age group of 20-34 years. Of the mothers, 259 (69.8%) were urban residents and 356 (96.0%) married. In this study, 106 (28.6%) of the mothers had no formal education and 345 (93.0%) were followers of the orthodox Christian religion. Of the mothers, 368 (99.2%) are Amhara in ethnicity and 230 (62.0%) are housewives (Table 1).

**Table 1:** socio-demographic characteristics of the mothers at FHCSH, Bahir Dar city, North West Ethiopia 2019, (n=371)

Variables	Categories	Frequency	Percent
Maternal age	<20	24	6.5
	20-34	275	74.1
	≥35	72	19.4
Residence	Urban	259	69.8
	Rural	112	30.2
Marital status	Married	356	96.0
	Single	12	3.2
	Divorced	3	0.8
Educational status	Had no formal education	106	28.6
	Primary	75	20.2
	Secondary	100	27.0
	Diploma and above	90	24.2
Religion	Orthodox	345	93.0
	Muslim	23	6.2
	Protestant	3	0.8
Ethnicity	Amhara	368	99.2
	Others*	3	0.8
Occupation	Housewife	230	62.0
	Government employee	70	18.9
	Merchant	38	10.2
	Daily laborer	21	5.7
	Others**	12	3.2

* Gurage, Agew, Oromo **private employee and student

### Obstetric characteristics of the mothers

Of the mothers, 205 (55.3%) were multipara and among them, 72 (35.2%) had < 24 months interpregnancy interval. In our study, 222 (59.8%) of the mothers had a four and above history of ANC visits, and 292 (78.7%) supplemented with had iron and folic acid. Of the mothers, 68 (18.3%) had a history of at least one BOH and among them, 34 (50.0%) had a history of spontaneous abortion. In their current pregnancy, 165 (44.5%) of the mothers have encountered at least one complication of pregnancy in the antepartum period, and PIH and PROM were the major complications encountered by 65 (39.4%) and 56 (33.9%) of the mothers respectively. Of the mothers, 143 (38.5%) had experienced complications during the intrapartum period, and non-reassuring fetal heartbeat pattern, prolonged labor, and malposition/malpresentation were the major complication encountered by 49 (34.3%), 47 (32.9%) and 39 (27.3%) respectively. Regarding the status of the pregnancy, 353 (95.1%) were singleton and, 274 (73.9%) of the mothers had spontaneous onset of labor. Of the mothers, 180 (48.5%) gave birth spontaneously, and 299 (80.6%) had a history of contraceptive method utilization (Table 2).

**Table 2:** obstetric characteristics of the mothers at FHCSH, Bahir Dar city, North West Ethiopia 2019, (n=371)

Variables	Categories	Frequency	Percent
Parity	Primi-Para	167	44.7
	Multi-Para	205	55.3
Inter-pregnancy interval (n=205)	< 24	72	35.2
	24-59	68	33.1
	≥ 60	65	31.7
Pregnancy status	Planned and wanted	326	87.9
	Unplanned but wanted	45	12.1
Number of ANC visit	< 4	149	40.2
	>= 4	222	59.8
Iron /folic acid supplement	No	79	21.3
	Yes	292	78.7
Duration of iron supplement in month (n=290)	<3	171	59.0
	>= 3	119	41.0
History of BOH	Yes	68	18.3
	No	303	81.7
Types of BOH (n=68) (multiple response possible)	Stillbirth	16	23.5
	Low birth weight	16	23.5
	Preterm delivery	6	8.8
	Congenital anomaly	2	2.9
	Spontaneous abortion	34	50.0
Complication in the antepartum period	Yes	165	44.5
	No	206	55.5
Type of pregnancy complication (n=165)	PIH	65	39.4
	APH	28	17.0
	PROM	56	33.9
	Amniotic fluid disorder	29	17.6
	Other *	6	3.6
Complication of labor	Yes	143	38.5
	No	228	61.5
Type of labor complication (n=143)	Prolonged labor	47	32.9
	Malposition and malpresentation	39	27.3
	Obstructed labor	9	6.3
	Non reassuring fetal heart rate pattern	49	34.3
	Other**	15	10.5
Status of the labor	Spontaneous	274	73.9
	Induced	97	26.1
Type of pregnancy	Single	353	95.1
	Multiple	18	4.9
Mode of delivery	SVD	180	48.5
	C/S	170	45.8
	Instrumental	21	5.7
Contraceptive used prior to current pregnancy	No	72	19.4
	Yes	299	80.6
Type of contraceptive used (n=299)	Depo-Provera	173	57.9
	Implant	54	18.0
	Intra uterine contraceptive device	5	1.7
	Oral contraceptive pills	62	20.7
	Emergency contraceptive	5	1.7

* abdomino- pelvic mass, IUGR and DVT **uterine rupture, CPD and failed induction

**Medical characteristics of the mothers:** in our study, 302 (81.4 %) of the mothers had no history of medical illness, while 69 (18.6%) has a history of medical illness. Among mothers who had a history of medical illness, malaria was responded to by 38 (55.1%) of mothers. Of the mothers, 301 (81.1%) had hemoglobin levels greater than 1g/dl, and 255 (68.5%) had a body mass index of 18.5-24.99 kg/m^2^ (Table 3).

**Table 3:** medical characteristics of the mothers at FHCSH, Bahir Dar city, North West Ethiopia 2019, (n=371)

Variables	Categories	Frequency	Percent
Medical illness	Yes	69	18.6
	No	302	81.4
Type of medical illness (n=69), (more than one answer is possible)	Malaria	38	55.1
	HIV	13	18.8
	Hypertension	10	14.5
	Other *	13	18.8
Maternal hemoglobin in g/dl	≤ 11	70	18.9
	>11	301	81.1
Maternal body mass index in kg/m2	<18.5	88	23.5
	18.5-24.99	255	68.5
	>25	28	7.5

Other * TB, heart problem, toxin nodular goiter, asthma and DM

### The proportion of fetal adverse birth outcomes

**Figure 1:**
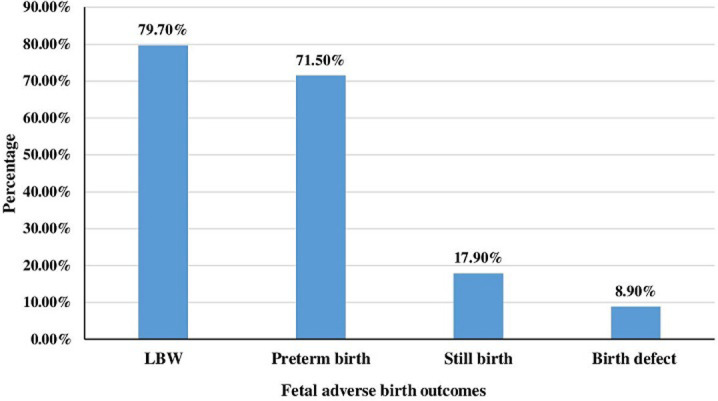
fetal adverse birth outcomes at FHCSH Bahir Dar city, North West Ethiopia 2019, (n=371)

This study finding showed that the proportion of FABO was 33.2% [95% CI: 28-38.5]. Among the FABO; 98 (79.7%), 88 (71.5%), 22 (17.9%), and 11 (8.9%) were LBW, preterm birth, stillbirth, and had visible birth defects respectively (Figure 1). The mean birth weight of the neonates was 2834 grams with a standard deviation of ± 618 grams, and the mean gestational age was 38.4 weeks with a standard deviation of ± 2.834 weeks.

### Factors associated with fetal adverse birth outcomes

In bivariate analysis: residence, educational status, number of ANC visits, iron supplementation, has a history of BOH, complications in the antepartum period, type of pregnancy, the onset of labor, has a history of medical illness and maternal hemoglobin level were candidate variables for multivariable analysis at P-value less than 0.20. In multivariable analysis mothers who are lives in rural area [AOR=4.37, 95% CI=2.44-7.83], < 4 ANC visit [AOR=1.91, 95% CI=1.08-3.40], has history of BOH [AOR=2.01,95% CI=1.03-3.93], complications in the antepartum period [AOR=4.32, 95% CI=2.44-7.65], has history of medical illness [AOR=2.44, 95% CI=1.25-4.79] and maternal hemoglobin < 11 mg/dl [AOR=4.63, 95% CI=2.40-8.93] were significantly associated with FABO at P-value less than 0.05 (Table 4).

**Table 4:** logistic regression analysis for FABO at FHCSH, Bahir Dar city, North West Ethiopia 2019, (n=371)

Variables	Fetal adverse birth outcome		COR (95%-CI)	AOR (95%-CI)	P-value
	Yes	No			
Residence					
Rural	61	51	3.80 (2.38-6.073)	4.37 (2.44-7.83)	0.001*
Urban	62	197	1	1	
Educational status					
Had no formal education	43	55	3.36 (1.73-6.51)	1.24 (0.48-3.21)	0.663
Primary	29	51	2.44 (1.23-4.91)	2.18 (0.85-5.57)	0.103
Secondary	34	69	2.12 (1.08-4.13)	2.41 (0.99-5.85)	0.051
More than secondary	17	73	1	1	
Number of ANC visit					
<4	63	86	2.28 (1.40-3.43)	1.91 (1.08-3.40)	0.027*
>4	54	168	1	1	
Iron supplementation					
No	35	44	2.04 (1.14-3.54)	1.57 (0.76-3.27)	0.225
Yes	82	210	1	1	
BOH					
Yes	32	36	2.07 (1.21-3.539)	2.01 (1.03-3.93)	0.041*
No	91	212	1	1	
Antepartum complication					
Yes	83	82	4.20 (2.65-6.66)	4.32 (2.44-7.65)	0.001*
No	40	166	1	1	
Onset of labor					
Induced	43	54	1.93(1.198,3.11)	1.80,(0.99,3.24)	0.051
Spontaneous	80	194	1	1	
Type of pregnancy					
Multiple	13	5	5.74(1.99,16.51)	1.97,(0.57,6.86)	0.284
Single	110	243	1	1	
Medical illness					
Yes	34	35	2.32(1.37,3.96)	2.44,(1.25,4.79)	0.009*
No	89	213	1	1	
Maternal hemoglobin level (g/dl)					
< 11	46	24	5.58(3.19,9.73)	4.63(2.40,8.93)	0.001*
>11	77	224	1	1	

* Significant at a P-value of <0.05.

## Discussion

This study finding showed that the proportion of FABO was 33.2% [95% CI: 28-38.5]. This figure was in line with studies conducted in Dessie referral hospital 32.5% [26], and Gamo Gofa Zone 37.6% [21]. However the result of this study was higher than the studies conducted in different parts of Ethiopia: like Gondar, Hosanna, Hawassa, and Shire, which ranges from 18.6%- to 24.5% [17-20], and it was also higher than a study conducted in Ghana 19% [31]. This variation might be since this study was done in a comprehensive specialized hospital, so the magnitude may rise because of an increasing number of referral cases from health centers, primary, and general hospitals. Mothers who had lived in rural areas were found to be four times higher odds of experiencing FABO than urban dwellers and this was consistent with the studies conducted in Gamo Gofa zone [32], southeast Ethiopia [33], and Northern Tanzania [34]. The reason might be due to poor access to quality pregnancy-related care in rural areas and a high load of work at home. It might also be linked to poor nutritional status, lack of early seeking of medical care, and long distance between their residence and health facility. Antepartum complications increased the odds of having FABO by 4.32 times. This finding was consistent with the studies done in Hosanna [17], Ardabil Iran [35], Tehran Iran [36], and China [9]. The reason may be explained in terms of the fact that the complications that have occurred during pregnancy have affected the health of the fetus in the uterus. This also might be related to the termination of pregnancy as a result of worsening of the complication before reaching full-term pregnancy and also due to the effect of complication, pregnancy was terminated without considering the birth weight.

Mothers with hemoglobin levels < 11 mg/dl have encountered 4.63 higher odds of experiencing FABO than those with hemoglobin levels greater than 11 mg/dl. The finding was in line with studies conducted in Dessie Ethiopia [26], Northern Tanzania [34], Nigeria [6], and Ghana [31]. The reason could be explained as the effect of anemia on the oxygen-bearing ability and its transportation to the placental site for the fetus. It might also be due to the causes of anemia during pregnancy in developing countries are multifactorial; because of various sociocultural problems like shortage of essential nutrients, iron folate, vitamin B12, C, and A, poverty, lack of awareness, poor dietary habits, parasitic infestation, blood loss, Human immunodeficiency virus, tuberculosis, malaria, too early pregnancies, high parity, short inter-pregnancy interval, cultural beliefs and practices, non-usage of insecticide-treated bed net and late booking of pregnant women at ANC [37-41]. On the other hand, anemia also affects impaired immune function, increasing the risk of cardiac diseases. On the other hand, mothers who had a history of BOH were two times more likely to experience FABO than their counterparts. This was consistent with the studies conducted in Shire [20], and Tanzania [15]. The reason could be explained as an adverse outcome in the first pregnancy of a woman living in the developing world is often a predictor of an adverse outcome in that woman's second pregnancy [42].

Mothers who had < 4 ANC visits were almost two times more likely to experienced FABO than those mothers who had > 4 ANC visits. The reason might be due to a lack of getting full services of ANC visits which includes early identification and management of any complications and prevailing diseases, health promotion, and care provision as well as birth preparedness and complication readiness plan. Furthermore, mothers who had a history of medical illness were found to be two times higher odds of experiencing FABO than mothers who did not have a medical illness. This finding was in line with studies done in Dessie [26], Hawassa [19], Tanzania [14,15]. This might be due to the presence of medical illness during pregnancy may affect the growth, development as well as well-being of the fetus. This study also identified that LBW and preterm birth are the major FABO. This finding was in line with studies conducted in different parts of the country [17-22].

**Limitation of the study:** clinician differences while determining the gestational age and hemoglobin level of some mothers who were determined after delivery were some of the limitations.

## Conclusion

In this study, the proportion of FABO was high compared to most of the studies done in Ethiopia. From the FABO: LBW and preterm birth cover the highest percentages. Place of residence, number of ANC visits, bad obstetric history, antepartum complications, medical illness, and maternal Hgb level are the predictors of FABO. Health education and counseling of pregnant mothers about nutrition, the importance of attending the recommended numbers of ANC visits, prevention and treatment of complications of pregnancy, early seeking of medical care, and management of antepartum complications are important elements to decrease FABO. Additionally, taking the supplemented iron and folic acid for three months before the first or next pregnancy and at least for three months during pregnancy are recommended to decrease FABO like a congenital anomaly.

### What is known about this topic

- Fetal adverse birth outcomes are in increasing mortality and morbidity of neonates and under-five children;
- The most common fetal adverse birth outcome are low birth weight and preterm birth.

### What this study adds

- We found that high prevalence of fetal adverse birth outcome at Felege Hiwot comprehensive specialized hospital;
- Factors associated with fetal adverse birth outcome at Felege Hiwot comprehensive specialized hospital;
- Integration of number of Antenatal care visit and fetal adverse birth outcome.
